# Electrochemiluminescence Detection of Sunset Yellow by Graphene Quantum Dots

**DOI:** 10.3389/fchem.2020.00505

**Published:** 2020-06-30

**Authors:** Huimin Niu, Xin Yang, Yilei Wang, Mingchen Li, Guangliang Zhang, Peng Pan, Yangyang Qi, Zhengchun Yang, John Wang, Zhenyu Liao

**Affiliations:** ^1^Tianjin Key Laboratory of Film Electronic and Communication Devices, Advanced Materials and Printed Electronics Center, School of Electrical and Electronic Engineering, Tianjin University of Technology, Tianjin, China; ^2^Tianjin Key Laboratory of Organic Solar Cells and Photochemical Conversion, School of Chemistry and Chemical Engineering, Tianjin University of Technology, Tianjin, China; ^3^Department of Materials Science and Engineering, National University of Singapore, Singapore, Singapore; ^4^Pony Testing International Group, Tianjin, China; ^5^Tianjin Food Safety Inspection Technology Institute, Tianjin, China

**Keywords:** graphene quantum dots (GQDs), electrochemiluminescence (ECL), sunset yellow, food safety, sensor

## Abstract

Use of food additives, such as colorants and preservatives, is highly regulated because of their potential health risks to humans. Therefore, it is important to detect these compounds effectively to ensure conformance with industrial standards and to mitigate risk. In this paper, we describe the preparation and performance of an ultrasensitive electrochemiluminescence (ECL) sensor for detecting a key food additive, sunset yellow. The sensor uses graphene quantum dots (GQDs) as the luminescent agent and potassium persulfate as the co-reactant. Strong and sensitive ECL signals are generated in response to trace amounts of added sunset yellow. A detection limit (signal-to-noise ratio = 3) of 7.6 nM and a wide linear range from 2.5 nM to 25 μM are demonstrated. A further advantage of the method is that the luminescent reagents can be recycled, indicating that the method is sustainable, in addition to being simple and highly sensitive.

## Introduction

In recent years, there have been growing concerns about food safety and the effects of certain food additives on human health in particular (Gan et al., 2013). Multiple studies have shown that excessive food additives, particularly synthetic colorants, often leads to cancer and other fatal effects, after a series of chemical reactions (Rovina et al., 2017). One common food additive, better known as FD&C Yellow No. 6 or sunset yellow, is a synthetic food colorant that is used widely (Vladislavić, 2018). Its strong and bright color, chemical stability, and low price have favored its use in the food industry (Zhang et al., 2010). However, the chemical functionalities of sunset yellow, i.e., its aromaticity and presence of an azo (N=N) moiety, can adversely affect human health if not controlled properly (Qiu et al., 2016; Sun et al., 2019). Food products with excessive added sunset yellow can cause allergies, anxiety, migraine, asthma, diarrhea, eczema, and other symptoms and can even lead to cancer (Yadav et al., 2012; Senthilkumar et al., 2013; Ding et al., 2019). Therefore, there is a clear need for a reliable technique that can accurately detect the amount of sunset yellow in food.

While there are already various methods for detecting sunset yellow, including high-performance liquid chromatography (HPLC) (Minioti et al., 2007; Alves et al., 2008; Sha et al., 2014), fluorescence spectroscopy (Yuan et al., 2016), UV-vis spectroscopy (Zou et al., 2013), and electrochemistry (Tran et al., 2019), these techniques are equipment-based and require properly trained operators and specialized equipment. Moreover, the detection limits are largely dependent on the operating conditions, and the overall costs are relatively high because organic solvents are required (Niu et al., 2013). A viable alternative to these existing approaches is electrochemiluminescence (ECL)-based detection, which has attracted much attention owing to simple instrumentation, operational convenience, low energy consumption, low environmental impact, and simple operation (Li S. et al., 2019).

ECL-based analysis uses electrochemically generated light to detect the presence of target analytes, similar to detection based on chemiluminescence and fluorescence (Liang et al., 2018). It has already been successfully employed for the detection of nitroaromatic, phenolic, and polycyclic aromatic compounds, among others (Zhang et al., 2014; Li S. et al., 2019). ECL reagents typically include luminol, ruthenium compounds, and quantum dots (Hao et al., 2017). In this study, we used graphene quantum dots (GQDs) as an ECL reagent to detect sunset yellow. GQDs are a class of quasi-zero-dimensional nanomaterials with diameters of <100 nm (Zhang et al., 2016). Owing to their small size, they have novel physical and chemical properties (Li et al., 2013). Compared with luminescent materials and other quantum dots, GQDs have higher specific surface area, water solubility, stability, and biocompatibility, along with other beneficial properties such as low physiological toxicity and ease of modification (Gan et al., 2013; Zhang et al., 2015; Liu et al., 2016). These advantages have led to the widespread application of GQDs in bio-imaging (Zhu et al., 2012), photoelectron devices (Gupta et al., 2011), photocatalysis (Gupta et al., 2015), and chemical sensors (Chen et al., 2018).

By applying GQDs to ECL, we can detect sunset yellow with high sensitivity. The ECL-based technique is more efficient and faster than other detection methods. Further, the reagent can be recycled and stored, and the luminescence image is stable. Moreover, it can also be used as a highly accessible and effective technique for the detection of other small molecular analytes in food sources (Ding et al., 2019).

## Experimental

### Reagents and Chemicals

GQDs were purchased from Nanjing XFNANO Material Technology Company. Sunset yellow was purchased from Shanghai Yuanye Biotechnology Co., Ltd. All other chemicals were purchased from Sigma-Aldrich. All the chemicals were used as received without further purification.

A 0.08 M KCl solution and phosphate-buffered saline (PBS) (0.01 M, pH 7) was used as the electrolyte, and 0.05 M K_2_S_2_O_8_ was used as the co-reactant.

### Apparatus

UV absorption spectra were measured on an Evolution 220 UV-vis spectrophotometer (Thermo Scientific). Fluorescence properties were evaluated on an F-7000 fluorescence spectrophotometer (Hitachi). Raman spectroscopy was performed at room temperature on a high-resolution laser confocal micro-Raman spectrometer (LabRAM HR Evolution, HORIBA). The morphology of the GQDs was characterized by transmission electron microscopy (TEM) and atomic force microscopy (AFM) using a Talos F200 X microscope (FEI) and an Evolution microscope (HORIBA JOBIN YVON S.A.S.), respectively. ECL measurements were conducted using an MPI-EII workstation (Xi'an Remax Electronic Science & Technology Co., Ltd., China).

### ECL Measurement Prcedure

ECL voltage curves were obtained using the MPI-EII workstation. Cyclic voltammetry (CV) was conducted using a conventional three-electrode system consisting of a glassy carbon electrode (GCE) (working electrode), Ag/AgCl in saturated KCl solution (reference electrode), and a platinum wire (counter electrode).

The electrode surface was pretreated before use. The surface of the GCE was polished with 0.3 and 0.05 μm Al_2_O_3_ on buckskin cloth and then rinsed with deionized water. All three electrodes were then sonicated for 5 min in ethanol and deionized water and dried at room temperature.

CV measurements were performed in 0.01 M PBS (pH 7) mixed with 0.08 M KCl as the supporting electrolyte and 0.05 M K_2_S_2_O_8_ as the co-reactant. For the ECL measurements, GQDs (0.1 mgmL^−1^, 200 μL) were mixed with a solution of 0.05 M K_2_S_2_O_8_ and 0.08 M KCl in PBS (1,800 μL) at a ratio of 1:9. The sunset yellow solutions of concentrations in the range of 2.5 × 10^−9^ to 2.5 × 10^−5^ M in PBS were prepared from a stock solution. PBS solutions from pH 3 to 11 were prepared to detect the influence of pH on luminous intensity. The effect of different values of pH on light intensity was detected with a sunset yellow concentration of 0.25 μM under the optimized conditions (GQDs, 0.1 mgmL^−1^; co-reactant, 0.05 M K_2_S_2_O_8_; supporting electrolyte, 0.08 M KCl; scan rate, 0.1 Vs^−1^). All of the experiments were performed within the potential range of −2.2 to −0.6 V.

## Results and Discussion

### Characterization of GQDs

**Figure 1:**
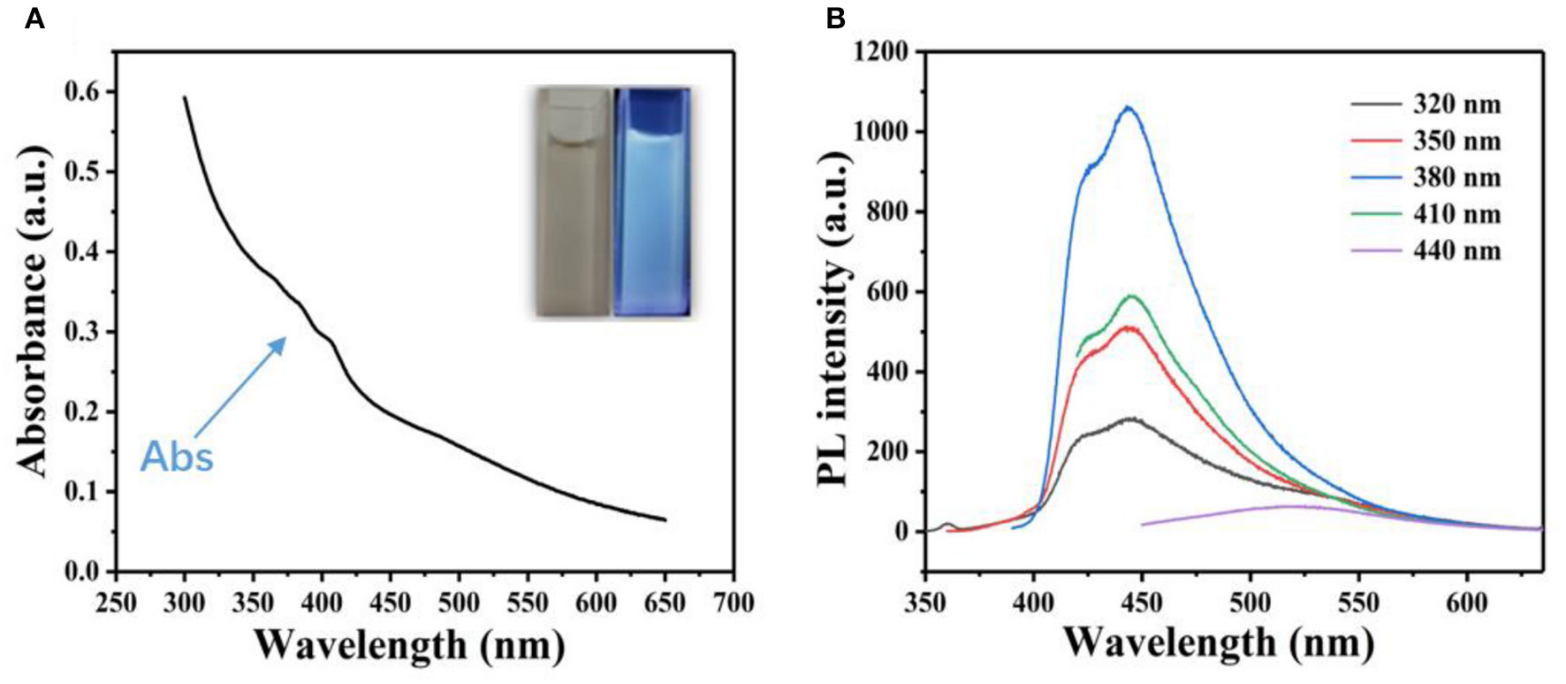
Spectrophotometric characterization of GQDs. **(A)** UV spectrum of an aqueous suspension of GQDs. Inset: aqueous suspension of GQDs under natural light (left) and blue fluorescence upon excitation by a 365 nm UV lamp (right). **(B)** Fluorescence spectra of GQDs at different excitation wavelengths.

The GQDs were characterized by UV-vis spectrophotometry (Figure 1A); a weak absorption peak was observed at 375 nm. The GQDs also exhibited photoluminescence (PL), as evidenced by the blue fluorescence emitted under UV illumination at 365 nm (Figure 1A, inset). The fluorescence spectra of the GQDs were obtained for different excitation wavelengths (Figure 1B). The emission wavelength of the GQDs was dependent on the excitation wavelength, which is consistent with the known characteristics of GQDs (Zhou et al., 2019). For example, when the excitation wavelength was increased from 320 to 440 nm, the PL peak is red-shifted (Zhou et al., 2009), with the strongest peak appearing at 450 nm upon excitation at 380 nm.

**Figure 2:**
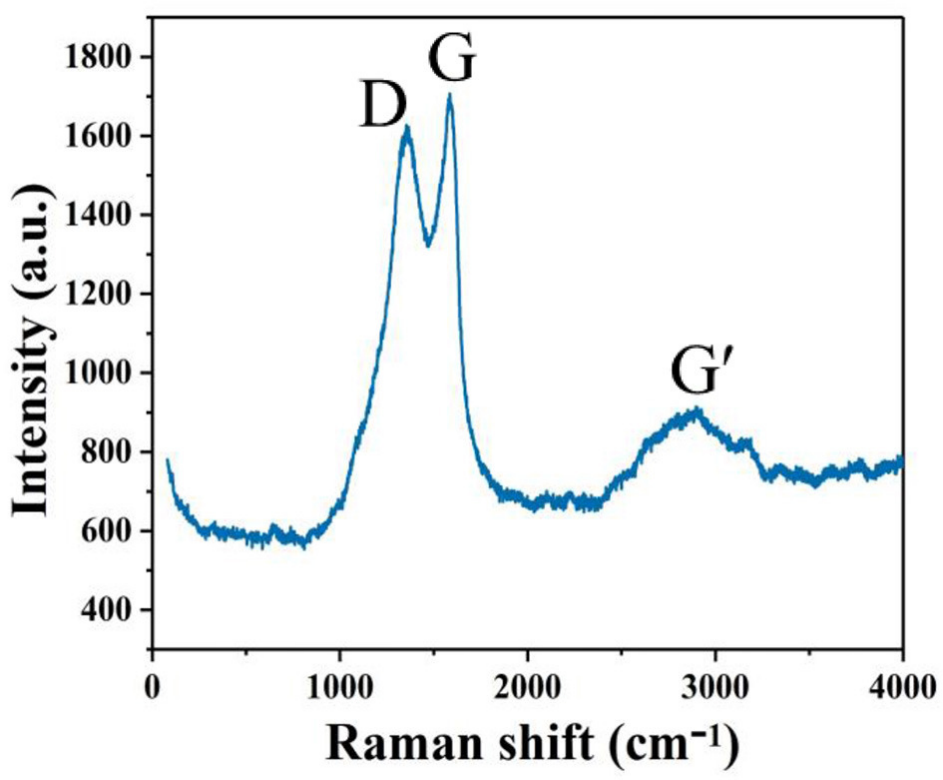
Raman spectrum of GQDs in an aqueous suspension.

The Raman spectrum of the GQDs shows G, D, and G' peaks (Figure 2); the strongest peak, referred to as the G peak (the main characteristic peak of graphene), which is attributed to the in-plane vibration of the sp^2^ carbon atoms, appeared at 1,583 cm^−1^ (Mishra and Bhat, 2019). The D peak observed at 1,353 cm^−1^ is generally considered the disordered vibration peak of graphene. There is an appreciable loss of the graphitic layered structure, as evidenced by the almost 1:1 ratio of the D and G band intensities. The weak broad band at ~2,896 cm^−1^, i.e., the G' peak (2D peak), is a two-fidelity resonance second-order Raman peak that characterizes a particular form of structure (interlayer stacking of carbon atoms) in graphene (Sun et al., 2019).

**Figure 3:**
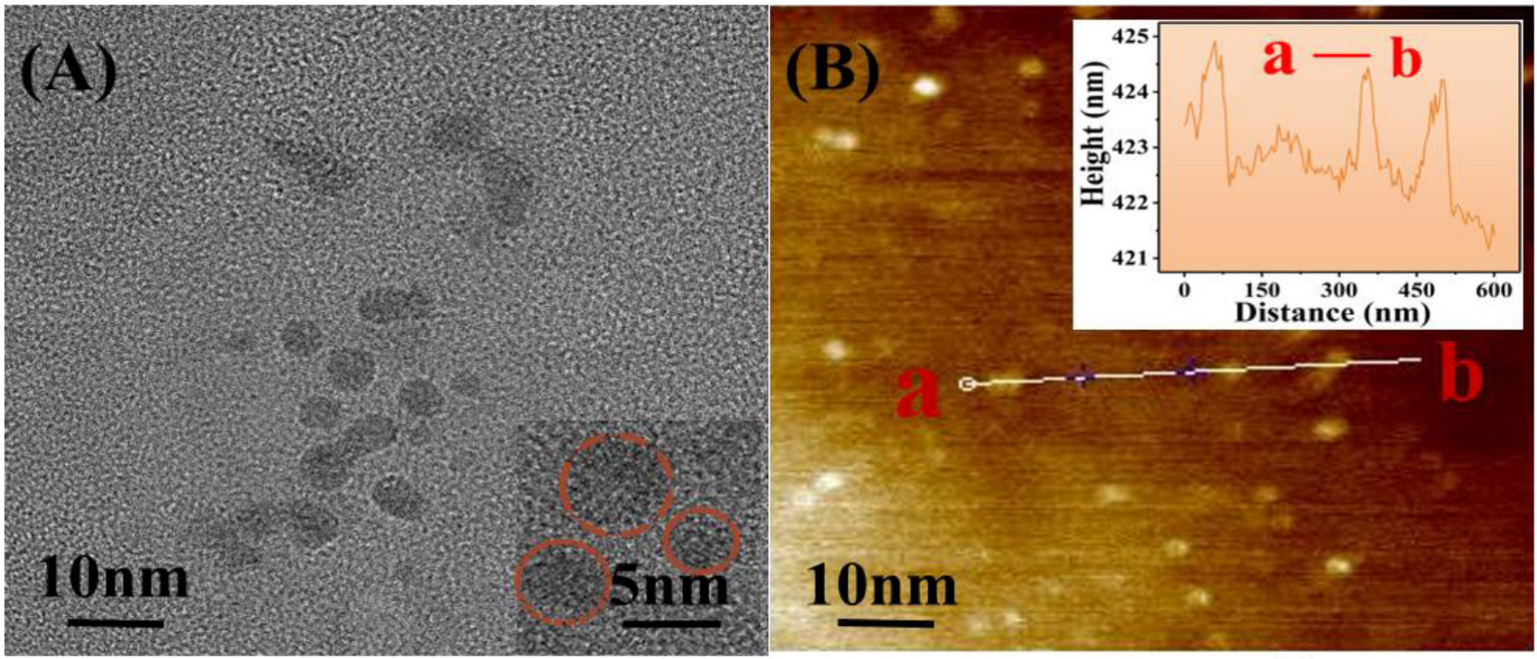
Morphological characterization of GQDs using TEM and AFM. **(A)** TEM image with the inset showing the GQDs at 2× magnification. **(B)** AFM image with the height profile of GQDs shown in the inset.

The morphology of the GQDs was then characterized using TEM and high-resolution AFM (Figure 3). TEM studies revealed that the GQDs are relatively uniform in size (average diameter = 5 nm) and are distributed in a single layer (Li et al., 2012). The crystal lattice of the GQDs can be clearly seen in Figure 3A (inset). From the AFM image (Figure 3B), the topographic heights of the GQDs were measured to be between 0.5 and 2 nm, with the average height being 1.2 nm (Figure 3B, inset), which suggests the presence of single or bi-layers in GQDs (Akilimali et al., 2020).

### Mechanism of the GQD Sensor to Detect Sunset Yellow

**Figure 4:**
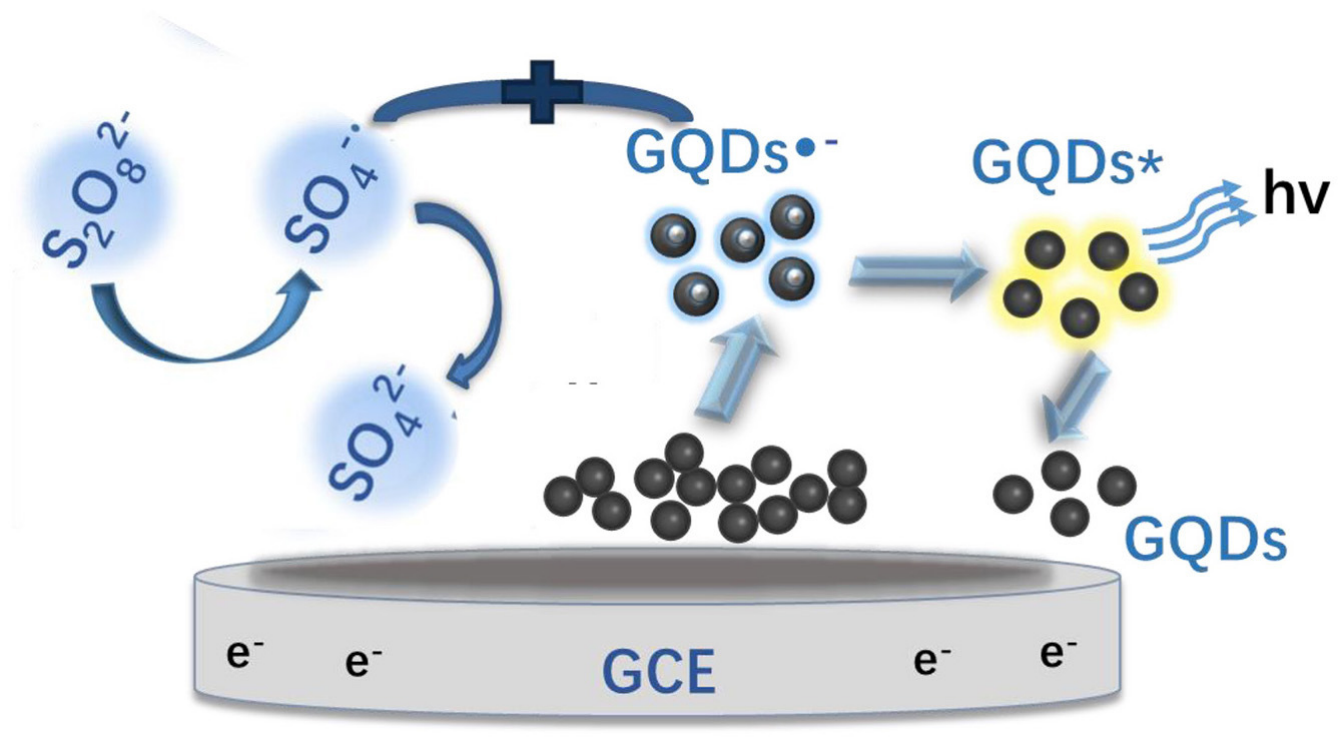
ECL mechanism of the GQD sensor. GQDs*: excited-state GQDs; h^+^: positive holes; GCE, glassy carbon electrode.

The ECL mechanism of the GQD sensor is schematized in Figure 4. Initially, reduction of K_2_S_2_O_8_ is performed to form ${\text{SO}}_{4}^{2-}$ and ${\text{SO}}_{4}^{-}$, while the GQDs are simultaneously reduced to anionic radical GQDs^−^. The strongly oxidizing SO4^−^ radicals react with the GQDs^−^ radicals via an electron-transfer annihilation process to produce the excited state of GQDs (GQDs^*^). Finally, the GQDs^*^ emit light and return to the ground state (Li et al., 2012; Hu et al., 2019; Li M. et al., 2019). As shown in Figure 7B, there is a very obvious ECL curve, and the ECL intensity is high enough. As shown in Figures 7A–C, when 0.025 μM sunset yellow is added, the current and the ECL intensity are clearly increased compared to when sunset yellow is not added. Sunset yellow was analyzed by the ECL signal of the GQD/K_2_S_2_O_8_ system, and the possible mechanism was proved through ECL and CV curves. Without adding sunset yellow, the electrons of the system come from the electrode. When sunset yellow is added, sunset yellow loses electrons and generates a new substance. The lost electrons are provided to the graphene quantum dot system, so GQD^*^ in the system increases (Gan et al., 2013). This process is described by Equation (1–5):

(1) $${\text{S}}_{2}{\text{O}}_{8}{}^{\text{2}-}\;+\;{\text{e}}^{-}\to SO_{4}{}^{2-}\;+\;SO_{4}{}^{•-}$$

(2) $$\begin{array}{r} \text{GQDs }+\;{\text{e}}^{-}\to GQDs^{•-} \end{array}$$

(3) $$\begin{array}{r} \text{GQD}{\text{s}}^{•-}\;+\;{\text{S}{\text{O}}_{4}}^{•-}\to GQDs^{*}+\;{SO_{4}}^{2-} \end{array}$$

(4) $$\begin{array}{r} \text{GQD}{\text{s}}^{*}\to GQDs\;+\;h\nu \end{array}$$

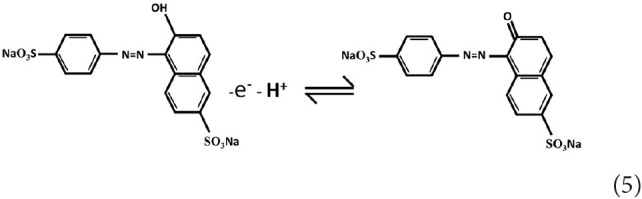


### Detection of Sunset Yellow by ECL Using GQDs

**Figure 5:**
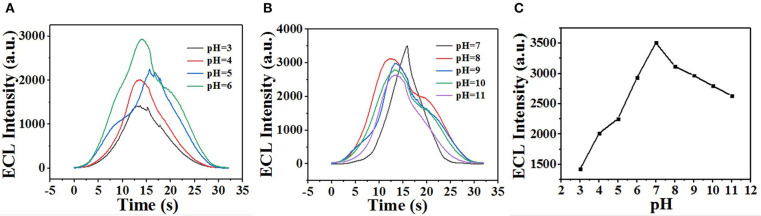
**(A,B)** Effect of pH on the ECL performance. **(C)** ECL intensity as a function of pH.

**Figure 6:**
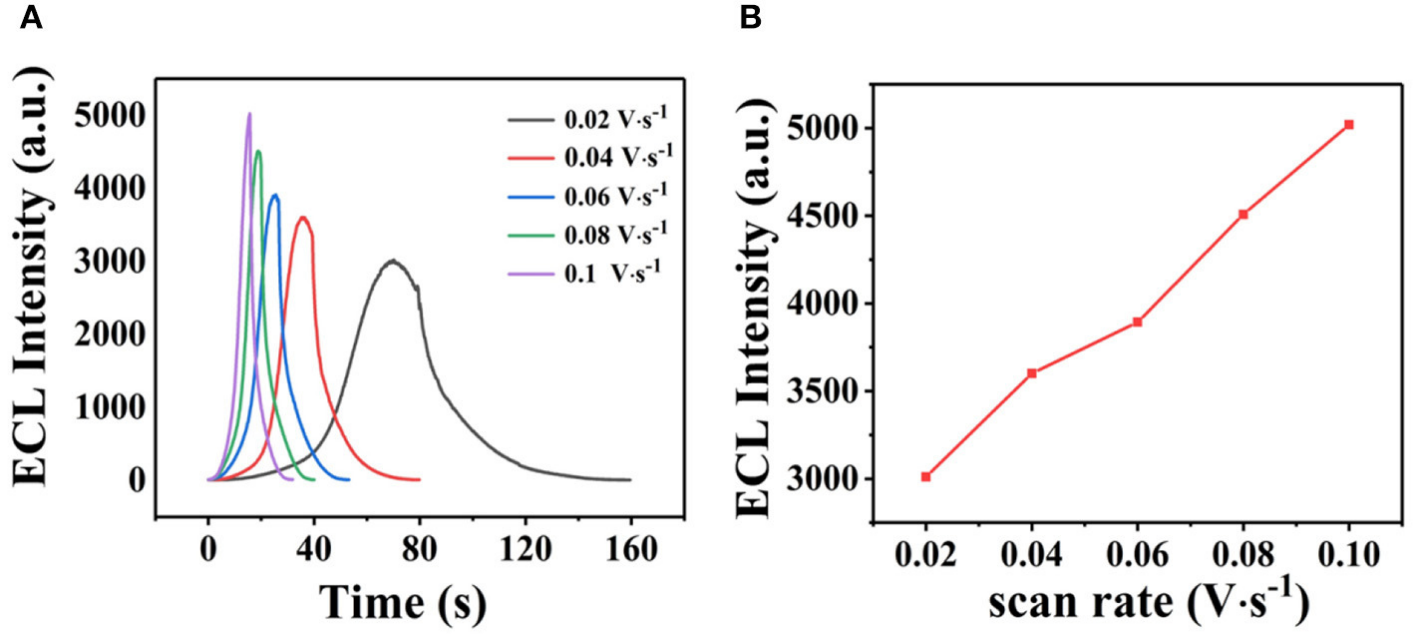
**(A)** Effect of scan rate on the ECL performance and **(B)** ECL intensity as a function of scan rate.

**Figure 7:**
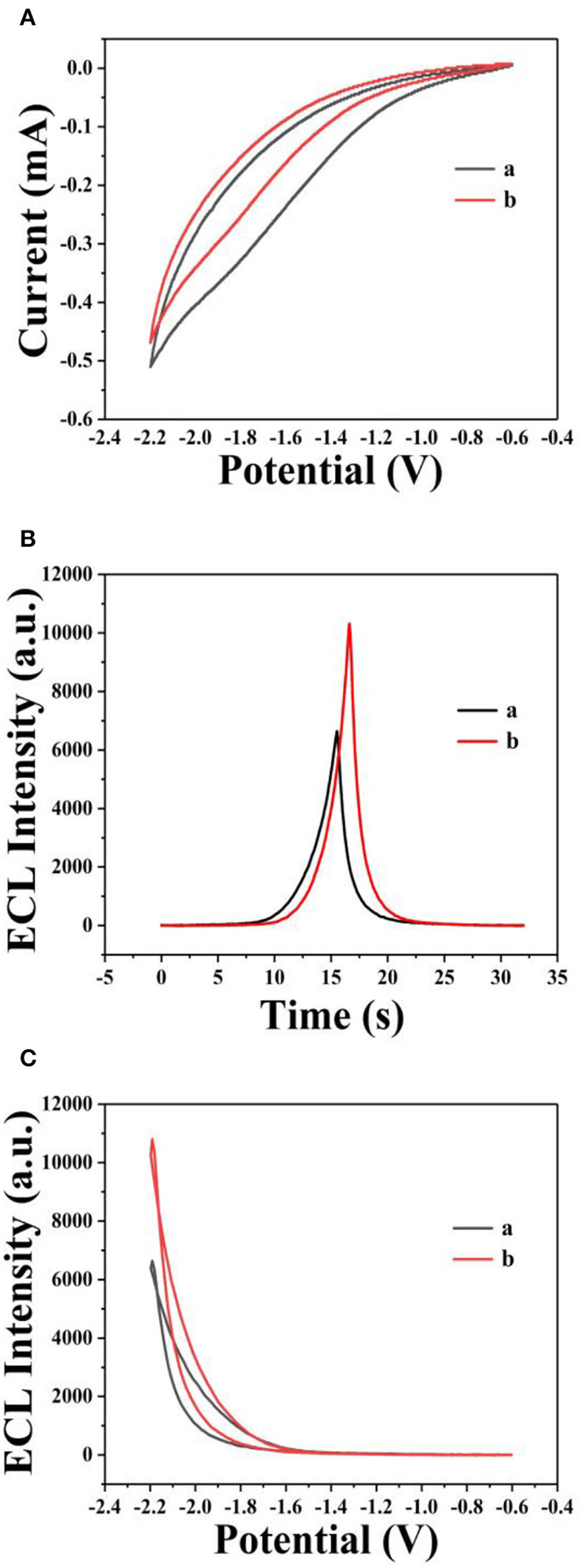
ECL responses from GQDs. Cyclic voltammograms **(A)** and ECL responses **(B)** of the GQDs/GCE electrode (a) without sunset yellow and (b) in the presence of 0.025 μM solution of sunset yellow. **(C)** ECL response of the GQDs/GCE electrode: (a) without sunset yellow and (b) in the presence of 0.025 μM solution of sunset yellow. Optimized conditions used for the reactions: GQDs, 0.1 mgmL^−1^; co-reactant, 0.05 M K_2_S_2_O_8_; supporting electrolyte, 0.08 M KCl; PBS (pH 7); scan rate, 0.1 Vs^−1^.

**Figure 8:**
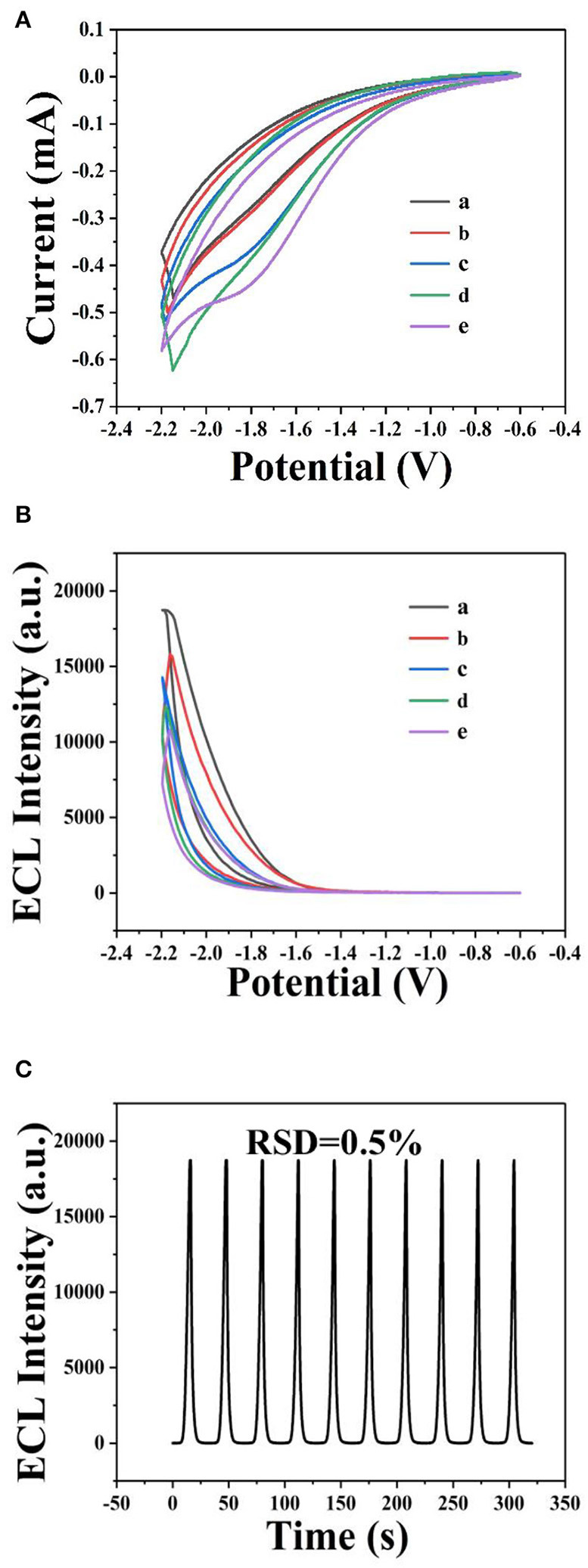
Detection of sunset yellow by ECL using GQDs. Cyclic voltammograms **(A)** and ECL response **(B)** of the GQDs/GCE electrode at different concentrations of sunset yellow: (a) 25 μM, (b) 2.5 μM, (c) 0.25 μM, (d) 0.025 μM, and (e) 0.0025 μM. **(C)** ECL responses of the biosensor to sunset yellow (25 μM) over 10 successive cycles. Optimized conditions used for the reaction: GQDs, 0.1 mgmL^−1^; co-reactant, 0.05 M K_2_S_2_O_8_; supporting electrolyte, 0.08 M KCl; PBS buffer (pH 7); scan rate, 0.1 Vs^−1^.

The ECL behavior of GQDs was investigated through CV using a cathodic co-reactant (K_2_S_2_O_8_). The scan rate in the CV method and the pH of PBS were optimized at room temperature to determine the best conditions for detecting sunset yellow. Figures 5A,B show the influence of pH from 3 to 11 on the ECL intensity. Figure 5C shows that the ECL intensity increases with pH in the range of 3 to 7 and then decreases at pH values beyond 7. When the pH was 7, the ECL intensity reached the maximum. Therefore, the optimal pH of PBS is 7 (Cheng et al., 2012). In addition, the ECL luminous intensity is different at different scan rates. Figures 6A,B illustrate that the ECL intensity strengthens in the range of 0.02–0.1 V•s^−1^, indicating that the excited-state substance is insufficiently produced at low scan rates. Therefore, the optimal experimental condition was a scan rate of 0.1 V•s^−1^ (Dai et al., 2010). The GQD system emits stably under the optimized conditions [GQDs, 0.1 mgmL^−1^; co-reactant, 0.05 M K_2_S_2_O_8_; supporting electrolyte, 0.08 M KCl; PBS buffer (pH 7); scan rate, 0.1 Vs^−1^]. The black and red curves in Figure 7A show the correspondences between the current and voltage in the absence and presence of 0.025 μM solution of sunset yellow, respectively. Upon adding sunset yellow, there was a significant increase in the ECL signal intensity (Figure 7B). Both the current and ECL signal intensity increased with increasing sunset yellow concentration (Figures 8A,B, respectively), indicating that the ECL system can differentiate the sunset yellow concentration. The stability of the ECL system is shown in Figure 8C.

**Figure 9:**
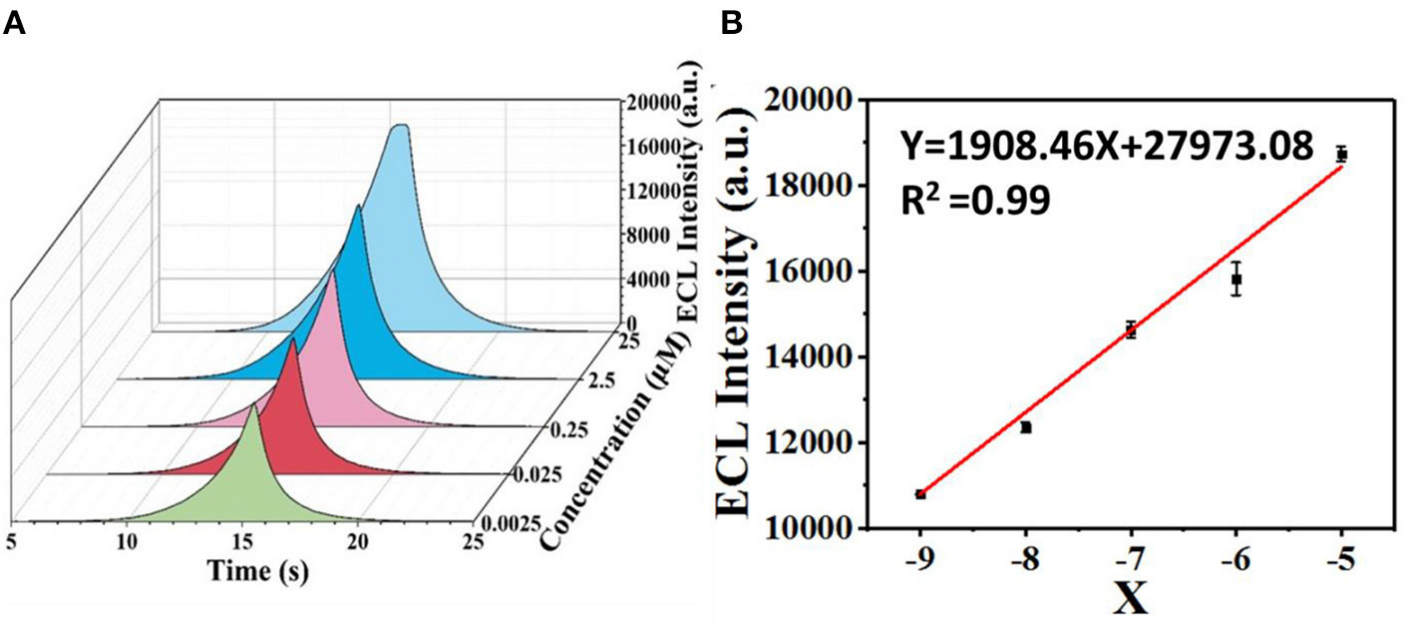
Determination of the limit of ECL detection of sunset yellow. **(A)** ECL intensities over time at different concentrations of sunset yellow. **(B)** Logarithmic calibration curve of the sunset yellow biosensor [X = Lg (C/2.5)].

Upon the addition of sunset yellow, the lost electrons are supplied to the system, generating the original system to produce more intermediate states. Therefore, the ECL signal increases with increasing concentration of sunset yellow in the range from 0.0025 to 25 μM (Figure 9A). A linear fit was obtained between the logarithm of the sunset yellow concentration and ECL intensity (Figure 9B), and the limit of detection (LOD) was determined to be 7.6 nM (signal-to-noise ratio = 3). A comparison between the system developed herein and previously reported sensors shows that this new sensor performs better than most existing sensors in the detection of sunset yellow (Table 1).

**Table 1:** Comparison of the system described herein with other reported methods used to detect sunset yellow.

**Electrode**	**Method**	**Linear range**	**LOD**	**References**
–	UV–vis	2–40 μgmL^−1^	–	Sorouraddin et al., 2011
–	HPLC	0.05–300ngmL^−1^	0.015 ngmL^−1^	Wu et al., 2013
–	Spectrometry	4.42–17.68 μM	193.0 nM	Dinç, 2002
–	Fluorescence	0.3–8.0 μM	79.6 nM	Yuan et al., 2016
Fe_3_O_4_@rGO/GCE	Electrochemistry	0.05–50 μM	50 nM	Han et al., 2014
Au/GO	Electrochemistry	0.01–3.0 μM	2.4 nM	Deng et al., 2016
ERGO/GCE	Electrochemistry	0.05–1.0 μM	19.2 nM	Tran et al., 2019
GO/MWCNTs/GCE	Electrochemistry	0.09–8.0 μM	25 nM	Vladislavić, 2018
MGO/β-CD/IL/AuNPs/GCE	Electrochemistry	0.005–2 μM	2 nM	Li et al., 2016
GQDs/GCE	ECL	0.0025–25	7.6 nM	This work

*rGO, reduced graphene oxide; GO, graphene oxide; MWCNT, multiwalled carbon nanotube; β-CD, β-cyclodextrin; AuNP, gold nanoparticle; ERGO, exfoliated reduced graphene oxide*.

### Selective Detection of Sunset Yellow by ECL Using GQDs

**Figure 10:**
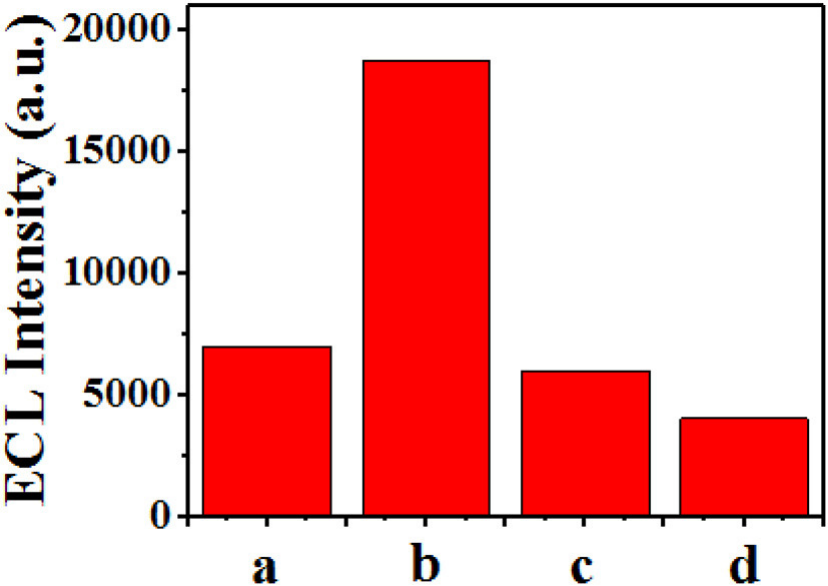
Demonstration of the selectivity of the ECL sensor. Response of the sensor when different substances were added: (a) none, (b) sunset yellow, (c) amaranth, and (d) sodium citrate.

Further, different substances, including sunset yellow, amaranth, and sodium citrate, were detected using the ECL sensor (Figure 10). Under the same experimental conditions, different detection substances of the same concentration (25 μM) were added, and the measured luminous intensity is shown in Figure 10. When sunset yellow was added, the light intensity increased significantly. However, when the other two substances were added, there was a small decrease in the light intensity. This result demonstrates that this method has preliminary selectivity in the detection of sunset yellow.

## Conclusions

Herein, we demonstrated a new, simple ECL sensor using GQDs that can be used to detect the food additive, sunset yellow. The ECL signal of the GQDs changes with the addition of sunset yellow. Under optimized conditions, the GQD sensor shows good linearity in the detection of sunset yellow in the concentration range of 0.0025–25 μM with a detection limit of 7.6 nM. Therefore, the method described herein is a highly sensitive one for detecting sunset yellow. This study also provides a basis for rapid screening for potentially harmful food additives.

## Data Availability Statement

All datasets generated for this study are included in the article/supplementary material.

## Author Contributions

HN, XY, and ML finished the material characterizations and electrochemiluminescence measurements. YW designed the photoluminescence characterizations. GZ and PP gave guidance about the electrochemical reactions. YQ analyzed the electrochemiluminescence mechanism. The manuscript was drafted by HN and ZY, while was revised by JW. ZL gave rise to the research meanings in the area of food safety. All authors critically revised the manuscript, approved the final version, and agreed to be accountable for all aspects of the work.

## Conflict of Interest

ZL was employed by the company Pony Testing International Group and Tianjin Food Safety Inspection Technology Institute in Tianjin, China. The remaining authors declare that the research was conducted in the absence of any commercial or financial relationships that could be construed as a potential conflict of interest.
